# Dynamic Robustness of Semantic-Based Collaborative Knowledge Network of Open Source Project

**DOI:** 10.3390/e23040391

**Published:** 2021-03-25

**Authors:** Shaojuan Lei, Xiaodong Zhang, Shilin Xie, Xin Zheng

**Affiliations:** School of Economics and Management, University of Science and Technology Beijing, Beijing 100083, China; b20180404@xs.ustb.edu.cn (S.L.); xieshilin@xs.ustb.edu.cn (S.X.); b20200433@xs.ustb.edu.cn (X.Z.)

**Keywords:** dynamic robustness, open source project, knowledge collaboration, weighted network, semantic-based

## Abstract

Robustness of the collaborative knowledge network (CKN) is critical to the success of open source projects. To study this robustness more comprehensively and accurately, we constructed a weighted CKN based on the semantic analysis of collaborative behavior, where (a) open source designers were the network nodes, (b) collaborative behavior among designers was the edges, and (c) collaborative text content intensity and collaborative frequency intensity were the edge weights. To study the robustness from a dynamic viewpoint, we constructed three CKNs from different stages of the project life cycle: the start-up, growth and maturation stages. The connectivity and collaboration efficiency of the weighted network were then used as robustness evaluation indexes. Further, we designed four edge failure modes based on the behavioral characteristics of open source designers. Finally, we carried out dynamic robustness analysis experiments based on the empirical data of a Local Motors open source car design project. Our results showed that the CKN performed differently at different stages of the project life cycle, and our specific findings could help community managers of open source projects to formulate different network protection strategies at different stages of their projects.

## 1. Introduction

In the open source design community, volunteers with diverse capabilities use the Internet to carry out innovation activities on open source products, either spontaneously or under the guidance of enterprises [1]. This collaborative design mode has significant advantages, such as low costs and high customer satisfaction in the aspects of product innovation, flexibility and foresight [2,3]. It has been successfully applied in fields such as open source software (e.g., Linux, Apache, Mozilla), knowledge sharing (e.g., Wikipedia, Baidu Encyclopedia), product design (e.g., Lego Mindstorms), among others. However, this self-organizing design model is easily affected by changes in the external environment, internal community management mechanisms, and other community competitions. This results in the degradation of designers’ collaborative behavior, which makes project progress slow and ineffective, and can even potentially cause the decline and end of the project [4,5]. Midha and Palvia [6] used both the popularity of a project and the amount of developer activity as indicators to explore the factors affecting the success of open source software. They found that the size of the community and the continuous innovation of personnel are fundamental for the continuous development and growth of the community. Griffith and Sawyer [7] posited that interactive collaboration is the best way to complete knowledge sharing, and Singh, Tan and Mookerjee [8] stated that participating members can improve development efficiency by relying on their own experience and collaboration with other members. Therefore, large-scale collaboration is an important way for community members to continue to innovate, which is also the main source of community success. Robustness refers to the ability of a system to maintain its original various functions when it faces the impact of changes in its internal structure or external environment [9]. In an open source project (OSP), the loss of designers and the weakening of designers’ willingness to collaborate on knowledge lead to the failure of collaborative behavior, which then affects the efficiency of the project. As such, it is of great practical significance both to identify the failure model of collaborative behavior in an OSP and to study the robustness of its collaborative knowledge network (CKN).

At present, research on OSPs can be roughly divided into two categories: (1) empirical and qualitative research, including the behaviors of participants, motivation for participation, and network structure characteristics in the community [10,11], thereby obtaining the operating rules and best practices of this type of community; and (2) the simulation modeling and experimental analysis of OSPs to conduct quantitative simulation research on its evolution mechanism, management mechanism, evolution trend, and so on [12,13]. An important method in both categories of research is the building of a social network model. Most OSP network models take (a) designers as nodes, (b) communication behaviors (such as email contact or mutual comments) as edges, and (c) frequency of communication as the weight [14,15,16,17]. For example, Bai and Deng [18] established a weighted network model based on the number of forwarded interactions between nodes in social network communities, to study the accuracy of social network link prediction. To study the influence that opinion leaders have on opinion followers in the open source community (OSC), Xu and Zhang [19] established a weighted CKN based on the number of comments between two designers. Li et al. [20] constructed a weighted idea transportation network between scientists in econophysics to study the impact of weight changes on the network. Toral, Martinez-Torres and Barrero [21] constructed a cooperation network based on the number of cooperation times between developers, then analyzed the cooperation behavior of existing developers in the OSC. Although the above network considered the weight of collaboration, the weight considered only the frequency of collaboration and not the content of collaboration. Different collaboration content also represents different collaboration intensity. Therefore, the constructed network was still an information dissemination network, which has not reached the level of the CKN. Consequently, a CKN based both on collaboration frequency and collaboration content can more accurately reflect the collaborative relationship among designers, which is the key of robustness analysis of OSCs.

The majority of research on network robustness focuses on the robustness of complex networks in real systems, such as in aviation, high-speed rail, public transport, supply chains, transportation and other fields [22,23,24]. As such, there are relatively few studies on the robustness of virtual networks without physical edges. Some scholars have studied the robustness of this type of network by constructing network models of different structures, mainly focusing on research on the research cooperation network and the Internet [25,26,27]. While research on the robustness of the OSP is still relatively lacking, the continuous development of the OSC has led to an increased recent focus here.

Research on network robustness is commonly based on the perspective of designer loss (i.e., node failure) [28,29,30]. For example, Frank et al. [31] measures the exit and entry of users according to the cost-benefit relationship of active nodes in the community (when the cost is higher than the benefit, the nodes leave the network), to studies the dynamic robustness of online social network based on the Tanaka, Morino and Aihara [32] studied the dynamic robustness of complex networks by attacking the nodes according to the node degree; They demonstrated that, in contrast to the structural fragility, the nonlinear dynamics of heterogeneously connected networks can be highly vulnerable to the failure of low-degree nodes. Zhang, Zhou and Hu [33] conducted node attacks on the OSC from both static and dynamic perspectives to analyze the robustness of the network. Fuge, Tee and Agogino [34] modeled a node attack simulation on the OpenIDEO community and found that an OSP with a core-edge structure has strong robustness. Tang and Liao [35] studied the robustness of the regional collaborative innovation network by removing nodes with high betweenness, in turn. These examples all use methods based on node failure (i.e., when the node is attacked, the node and the connected edges are deleted).

However, in the OSP, rather than designers directly exiting from the project when they are negatively affected, they would first decline the collaboration intention with other designers. The failure mode of network robustness analysis should reflect this process more accurately by changing the node failure mode to an edge failure mode. In addition, existing robustness research tends to be static [36,37,38,39] as it only analyzes the network in one particular time period, but in the development process of an OSP, the collaborative network has different structural characteristics at different stages. Therefore, the dynamic robustness of the network should be studied from the perspective of dynamic evolution (i.e., at each stage of the network).

We summarize the current research on network model and robustness, as shown in Table 1. This paper proposes a method to assess the dynamic robustness of CKNs based on semantic analysis and edge failure mode correspondingly. The remainder of the paper is structured as follows. In Section 2, we construct a network with comprehensive collaboration information using (a) OSC designers as network nodes, (b) collaborative behaviors among designers as edges, and (c) the collaborative text content intensity and collaborative frequency intensity as the edge weights. To study the robustness of the network from a dynamic viewpoint, we also construct CKNs from three different stages of the project life cycle: the start-up, growth and maturation stages. In Section 3, we use the connectivity and collaboration efficiency of the weighted network as robustness evaluation indexes. The specific calculation method is also presented. In Section 4, we design four edge failure modes based on the behavioral characteristics of OSP designers: (1) collective failure of knowledge contribution behavior, (2) successive failure of knowledge contribution behavior, (3) collective failure of knowledge dissemination behavior, and (4) successive failure of knowledge dissemination behavior. In Section 5, we conduct dynamic robustness analysis experiments based on the empirical data of a Local Motors OSP for a car design. Finally, in Section 6, we present suggestions for network robustness protection based on our results.

## 2. Construction of Weighted Collaborative Knowledge Network

### 2.1. Semantic-Based Weight Calculation

The OSC makes use of public knowledge and creativity to enable a large number of participants to share, suggest, evaluate, and improve knowledge with other participants through the Internet [40,41,42]. The collaborative behavior of designers includes: (1) knowledge-level behavior, which is measured by the content of the collaboration between designers; and (2) non-knowledge-level socialized behavior, which is represented by the frequency of the collaboration. Despite the content and frequency of collaboration being two key factors when constructing a CKN, most studies on OSC networks consider only the frequency of collaboration when calculating the weight of the network. This cannot truly reflect the collaboration intensity between designers in the OSC.

We constructed a semantic-based CKN by taking OSC designers as nodes, collaborative behaviors between nodes as edges, and collaborative content intensity and collaborative content frequency as edge weights. The network edge weight $\;W_{i,j}$ is obtained by weighting the collaborative content intensity $\;g_{i}$ and the collaborative frequency intensity $k_{i,j}\;$between designers, as shown in Equation (1):

(1) $$W_{i,j}=\alpha g_{i}+\beta k_{i,j},$$

where $\;g_{i}\;$is obtained by calculating the matching degree between the collaborative content and the project keywords in order to normalize it $k_{i,j}$ is obtained by normalized processing of the collaboration times between designer *i* and designer *j*, and *α* and *β* are the influence coefficients of the content intensity and frequency intensity, respectively, satisfying *α* + *β* = 1. The following focuses on the calculation of the collaborative content intensity $g_{i}$.

First, keywords are extracted from the project’s overall text comments using the RAKE algorithm [43]. Then, the value of $\;g_{i}$ is calculated according to the matching degree of these keywords. The specific calculation steps are as follows:

1. Word segmentation: Take all collaborative content contained in the community project as the target text, then take punctuation marks and stop words as word segmentation intervals to obtain a candidate set of text keywords, $T=\left\{t_{1},\;t_{2},\;t_{3},\ldots ,t_{z}\;\right\}$.
2. Construct the co-occurrence matrix, as shown in Equation (2):
  (2) $$D_{zz}=\left[\begin{array}{ccc} a_{1,1} & \cdots & a_{1,z}\\ \vdots & \ddots & \vdots \\ a_{z,1} & \cdots & a_{z,z} \end{array}\right]$$
  where $\;a_{m,m}$ is the frequency of the occurrence of the candidate word in the text, and $a_{m,n}$ is the frequency of the co-occurrence of the candidate words *t_m_* and *t_n_* in the same phrase.
3. Calculate candidate word weight: According to the co-occurrence matrix, get the degree of candidate word $t_{m}$: $Deg_{m}\;=\overset{z}{\underset{n=1}{\sum }}a_{m,n}$, the frequency of candidate word $\;t_{m}\;$:$\;Feg_{m}=a_{m,m}$ then use their ratio to represent the weight $W_{t_{m}}$ of the candidate word $t_{m}$ as shown in Formula (3):
  (3) $$W_{t_{m}}=\frac{Deg_{m}}{Feg_{m}}=\frac{\sum_{n=1}^{z}a_{m,n}}{Feg_{m}}.$$

Arrange in descending order according to the calculated weight $W_{t_{m}}$ take the candidate words in the top 1/3 of the ranking as the keywords of the text, and output the keyword set $T^{\prime }$, and the weight value $W_{t_{m}}$ of each keyword.

For example, by calculating the weights according to the content of the project reviews of an open source car community, the top ten keywords in the weight ranking can be obtained, as demonstrated in Table 2.

4 Calculate the content intensity of each designer: Follow step (1) to segment the overall comment content of each designer in the project to obtain a set of keyword candidates for each designer, $\;T_{i}=\left\{t_{1},\;t_{2},\;t_{3},\ldots \right\}$, then calculate $g_{i}{}^{\prime }$ as the sum of the weights of the keywords contained in the designer comment text, as shown in Formula (4):
  (4) $$g_{i}{}^{\prime }=\sum_{t_{m}\in T^{\prime }\cap T_{i}}W_{t_{m}},$$
  where $T^{\prime }$ is the keyword set, $T_{i}\;$is the candidate word set of designer *i*, and $t_{m}$ is the keyword contained in designer *i*. For example, Table 3 shows a list of the top ten nodes in the content intensity of an open source car community.
5 Obtain $g_{i}$ by normalizing $\;g_{i}{}^{\prime }$.

### 2.2. Network Structure Characteristics in Different Life Cycle Stages

Life cycle theory refers to the process of birth, growth, aging, illness and death of a certain thing. This concept coincides with the development of the OSC, and some scholars have introduced life cycle theory into virtual network research [44,45]. For example, Moingeon, Quelin and Dalsace [46] divided virtual communities into “formation stage, development stage, institutionalization stage and decline stage” by referring to the three-stage model of traditional organization development, and Tan et al. [47] divided the life cycle of the community into four stages (initial, growth, maturity and decline) to study the cooperative behavior choices of virtual academic communities. However, existing research on the dynamic robustness of the OSC usually only analyzes the network at a single static time node rather than taking into account specific or multiple life cycle stages. For a more accurate representation of robustness, it is necessary to analyze the characteristics of the CKN of OSC projects over time.

In this paper, we used Local Motors, an OSC for car design, as the research object. Local Motors has the world’s largest automotive design communication community, and the online community is active with more than 8000 automotive design enthusiasts from 121 countries. This community has an avant-garde design concept of “production for customers”, where designers can freely choose design projects of interest, exchange creative models, and propose design solutions. It is a typical open source community with the characteristics of open source, large-scale collaboration, product innovation, and dynamics. It can represent most open source communities, and the research results and corresponding management strategies can be applied to other communities with universal applicability.

This paper analyzes the impact of the failure of knowledge collaboration behavior on robustness from a micro perspective. In order to make the research results more specific and easier to apply to other networks, we chose project LF-01, which has the largest number of participants and can best reflect the characteristics of this community, for our research. This project was established in January 2014, and as of 16 November 2016, the project contained 673 designers and 7757 instances of communication. The selection of this project has the following considerations. (1) We compared all the projects in the Local Motor community and found that as of 16 November 2016, “LM SF-01” has the largest number of participants, which best reflects the characteristics of the community. (2) The network features of LF-01 are consistent with most projects; it can therefore suitably represent the situation of most OSPs. (3) Selecting a single project for analysis can clearly describe the evolution process of project robustness from a microscopic point of view.

**Figure 1:**
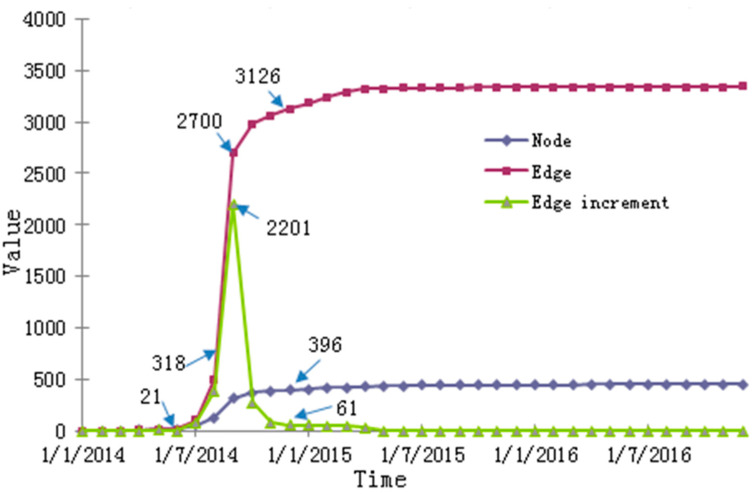
Scale evolution diagram of the collaborative knowledge network of LF-01 project.

Python3.6 software was used to crawl all collaboration data of LF-01, from creation to end, and the evolution process of the project was analyzed on a timeline for the number of nodes, edges and incremental edges. As Figure 1 shows, this evolution curve demonstrates (a) an initial trend of slow growth, followed by rapid growth, then slow growth, and finally stability, and (b) an increase then a decrease of the incremental edges.

As Figure 1 shows, the evolution curve of nodes and connecting edges is basically consistent with the product life cycle theory. Therefore, as per the growth rate method in the product life cycle theory, we dividedLF-01′s network into three stages: (1)the start-up stage (prior to 10 June 2014),where the growth rate of nodes and edges in the network is slow, and the value of nodes and edges is at its minimum; (2) the growth stage (from 10 June 2014 to 10 September 2014), where (a) the network nodes and connecting edges have the fastest growth rate, and (b) on 10 September 2014, the nodes and connecting edges appear to be at a relatively obvious inflection point, and their growth rates begin to slow; and (3) the maturation stage (after 10 December 2014), where the nodes and edges have the slowest growth rate but the highest value. For the purpose of simple description, these three time nodes (10 June 2014; 10 September 2014; and 10 December 2014) are respectively called the start-up stage, the growth stage and the maturation stage of the network. For these three stages, we constructed a semantic-based weighted CKN. Here, the intensity of collaborative content, α, and collaborative frequency, β, were considered equally important, and were each given a value of 0.5. Table 3 shows some of the network topology parameters of the network during the three periods.

Table 4 shows that small-world, scale-free and disassortative characteristics are present in all three network stages. The small-world characteristic shows that the network has some “shortcut” connections to connect different subgroups, that is, there are some key cooperation in the knowledge collaboration relationship of many participants, and they play a key role in reducing the network distance. The scale-free characteristic indicates that during the evolution of the project network, new participants tend to connect to larger nodes in the original network. The disassortative characteristic reflects that nodes with lower degree values are more inclined to establish connections with nodes with higher degree values. Since the weighted CKNs of all three stages have small-world, scale-free and disassortative characteristics, the outflow of designers may bring about serious consequences. This indicates that the robustness analysis of these networks is very necessary.

## 3. Robustness Evaluation Index

It is generally believed that the robustness of the network is the degree of retention of network performance when network nodes or edges fail [49,50,51]. For the CKN of an OSC, the impact of network node or edge failure mainly includes two aspects: (1) the network connectivity is destroyed, which reduces the collaborative knowledge intensity; and (2) the collaborative knowledge efficiency decreases, which increases the difficulty of collaborating knowledge to the network. Therefore, the robustness evaluation index proposed in this paper also includes two aspects: network connectivity and collaborative knowledge efficiency.

### 3.1. Relative Size of Network Connectivity S

Connectivity is an important performance index of the network, which is usually expressed by the relative size of the most connected subgraph. It refers to the proportion of the number of nodes in the subnet with the largest number of nodes to the number of all remaining nodes after the network is attacked. However, the relative size of the most connected subgraph cannot reflect the impact of the change of knowledge collaboration behavior on network performance. In this paper, the relative connectivity size, *S*, is defined as the relative size of the largest connected subgraph node intensity of the network, so as to reflect the degree of network connectivity retention:

(5) $$S\;=\frac{S_{lc}^{\prime }}{S_{lc}},$$

where $S_{lc}^{\prime }$ is the sum of the node intensity of the maximum connected subgraph of the network after being attacked, and $S_{lc}$ is the sum of the node intensity of the original network. The calculation formula for the sum of node intensity is

(6) $$S_{lc}=\sum_{i\neq j}^{N}w_{ij},$$

where *N* represents the total number of nodes in the network, and $w_{ij}$ represents the edge weight of nodes *i* and *j*. In the weighted CKN of the OSC, the node intensity represents the collaborative knowledge intensity. Therefore, the smaller the value of *S*, the greater the decrease in the collaborative knowledge intensity after the network is attacked, that is, the less robustness of connectivity, and vice versa.

### 3.2. Relative Size of Collaborative Knowledge Efficiency H

Network efficiency indicates how easy it is for collaborative information to enter the network. It is also an important measure of network performance. The network efficiency is expressed as the sum of the efficiency of all nodes in the network, where node efficiency is the reciprocal of the shortest path length between two nodes [13]:

(7) $$E=\frac{1}{n\left(n-1\right)}\sum_{i\neq j}\frac{1}{d_{ij}},$$

where $d_{ij}\;$is the distance between nodes *i* and *j*, and *n* is the number of nodes.

Although the above network efficiency formula describes the difficulty of information dissemination, it does not reflect the weighted characteristics of the CKN. Therefore, this paper defines collaborative knowledge efficiency by referring to network efficiency formula *E_G_*:

(8) $$E_{G}=1-\frac{1}{n\left(n-1\right)}\sum_{i\neq j}\frac{1}{w_{d_{ij}}},$$

where $w_{d_{ij}}$ is the sum of weights on the shortest path between nodes *i* and *j* in the CKN.

Furthermore, the relative size of collaborative knowledge efficiency, *H*, is used as another important index to measure the robustness, so as to reflect the degree of retention of collaborative knowledge efficiency after the network is attacked:

(9) $$H\;=\frac{E_{G}{}^{\prime }}{E_{G}},$$

where $E_{G}{}^{\prime }$ is the collaborative knowledge efficiency of the attacked network and $E_{G}\;$is the collaborative knowledge efficiency of the original network. Obviously, the value range of *H* is [0,1]. When *H* = 0, it indicates that the network efficiency drops to its lowest after the attack, that is, designers in the network do not have any form of cooperation. When *H* = 1, it indicates that the efficiency of the whole network remains at the original level, without any impact on the network efficiency due to the failure of edge weights.

## 4. Failure Mode Design of the Robustness Analysis

The design of failure modes is the key to robustness analysis. Most previous research is based on the failure of nodes themselves, and little research exists on the failure of collaborative knowledge behavior between nodes.

In OSPs, collaborative knowledge behavior can be divided into knowledge contribution behavior and knowledge dissemination behavior. The edge weight of the CKN constructed in this paper is weighted based on the collaborative content intensity and collaborative frequency intensity, so it can effectively describe the knowledge contribution behavior between nodes. The order of knowledge contribution behavior between nodes can be reflected by the order of edge weights. In addition, the number of edge betweenness in the constructed network is the number of times that the edge acts as the intermediary in the network, where “intermediary” means that the edge occupies a key connection position in the dissemination path of the network. This is an important parameter to measure knowledge dissemination, which can describe the behavior of knowledge dissemination between nodes. If the order is based on the number of edge betweenness, it can reflect the order of collaborative knowledge behavior intensity between nodes.

Considering the above two kinds of collaborative knowledge behavior, as well as the two scenarios of successive failure occurrence and collective failure occurrence, we designed four failure modes for collaborative behavior, as shown in Table 5.

## 5. Dynamic Robustness Analysis Experiments

Based on the collaborative knowledge behavior and failure modes of the network constructed in Section 4, we used Python3.6 software to program the simulation of the robustness index changes of the CKN under each of the four regular failure modes (BS, BC, WS, WC) and the random failure mode (R) of the start-up, growth, and maturation stages.

### 5.1. Robustness Analysis during Project Start-Up Stage

**Figure 2:**
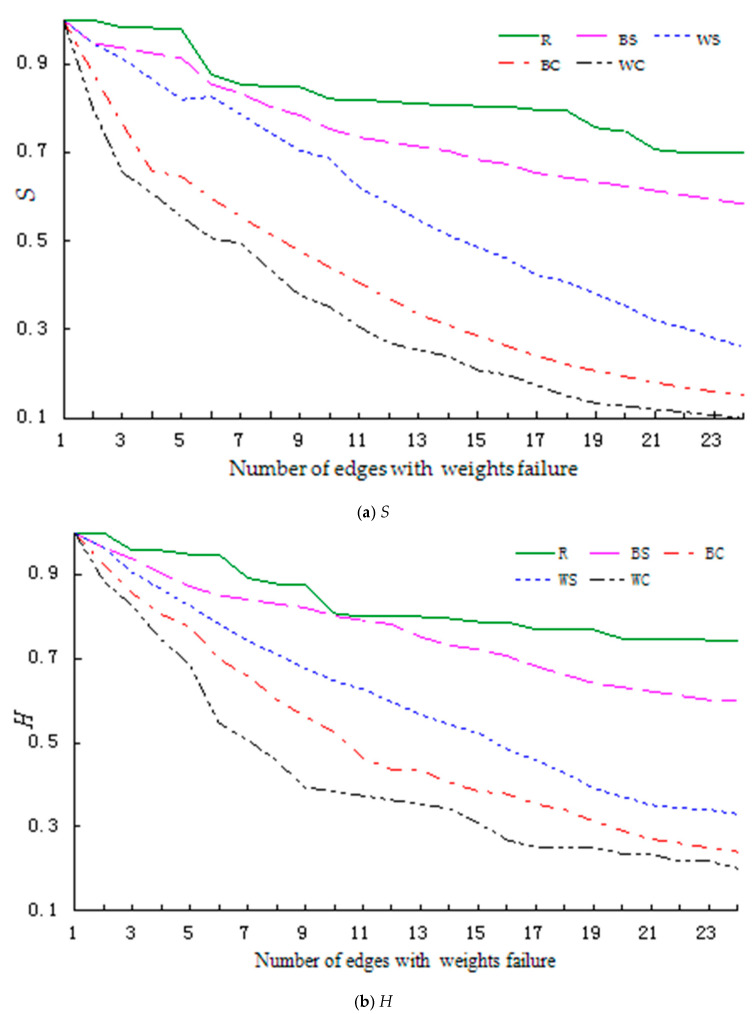
Changes in the robustness index of the network during the start-up stage (Legend number of edges: actual number of edges = 1:5). (**a**) shows the changes in relative size of network connectivity (*S*). (**b**) shows the changes in relative size of collaborative knowledge efficiency (*H*).

The robustness index changes during the start-up stage are shown in Figure 2.

Figure 2 shows that the decline rate of the index value is significantly higher for the four regular failure modes (BS, BC, WS, WC) than for the random failure mode (R). Further, the decline rate is higher in failure modes for (a) knowledge contribution behavior (WS, WC) as opposed to knowledge dissemination behavior (BS, BC), and (b) collective failure (BC, WC) as opposed to successive failure (BS, WS). When we performed a paired T-test on this data, the index values of the network under different failure modes were significantly different. See Table 6 for part of this test result.

Based on the above analysis, we can conclude that when the network is in the start-up stage, the random failure mode has the highest robustness, followed by the successive failure of knowledge dissemination behavior mode, successive failure of knowledge contribution behavior mode, collective failure of knowledge dissemination behavior mode, and collective failure of knowledge contribution behavior mode, namely R > BS > WS > BC > WC.

Paired sample T-test for different failure modes of network in start-up stage (*α* = 0.05) Table 6 shows that under the failure modes for knowledge contribution behavior(WS, WC), the values of Sand H decrease to 0.5 when 5 edges collectively fail (WC), whereas 15 edges need to fail to achieve the same effect under the successive failure mode (WS).Similarly, under the failure modes for knowledge dissemination behavior (BS, BC), the values of S and H decrease to less than 0.3 as the number of failure edges increases under the collective failure mode (BC), whereas these values remain much higher under the successive failure mode (BS).

In the start-up stage, the increase in collaborative knowledge behavior greatly improves the robustness of the network. Therefore, in addition to effectively promoting a project in the start-up stage, community managers should also develop corresponding incentive mechanisms to increase knowledge sharing behavior by (a) encouraging designers who have already entered the community to introduce more designers to the community and (b) encouraging existing designers to share more of their knowledge.

### 5.2. Robustness Analysis during Project Growth Stage

**Figure 3:**
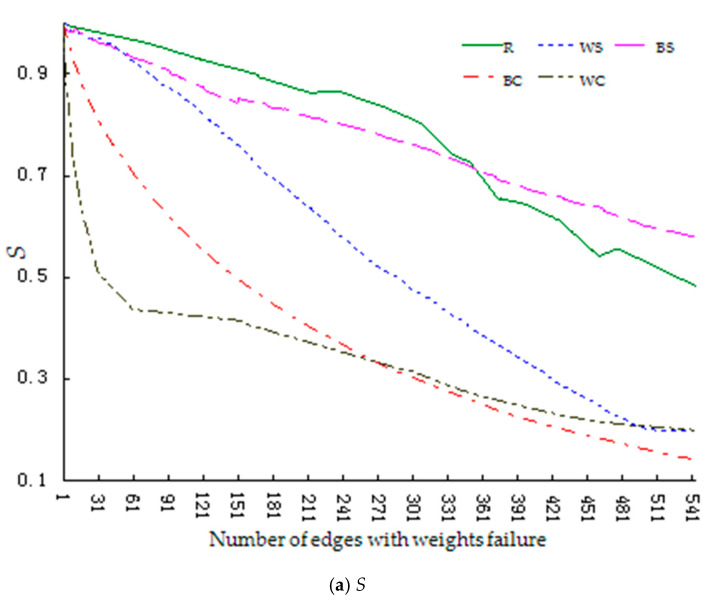

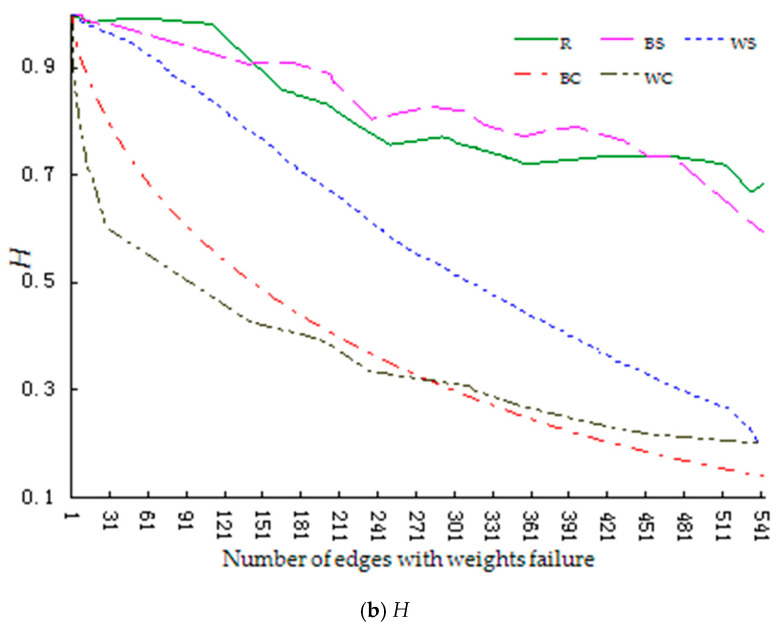
Changes in the robustness index of the network during the growth stage (Legend number of edges: actual number of edges = 1:5). (**a**) shows the changes in relative size of network connectivity (*S*). (**b**) shows the changes in relative size of collaborative knowledge efficiency (*H*).

The robustness index changes during the growth stage are shown in Figure 3.

Similar to the start-up stage, Figure 3 shows that the decline rate of the index value in the growth stage is significantly higher when the network faces regular failure (BS, BC, WS, WC) rather than random failure (R). Further, the decline rate is slower in the failure mode for successive knowledge dissemination behavior (BS) than in the other three regular modes (BC, WS, WC), instead following the same trend as the random failure mode (R).The descending magnitude of the curve under the other three regular failure modes is: collective failure of knowledge contribution behavior (WC) > collective failure of knowledge dissemination behavior (BC) > successive failure of knowledge contribution behaviors (WS).

We further test the significant difference of each index. Since we focuses on the performance changes of the network under different failure modes in order to propose more effective solutions. Therefore, the process data with the values of each index dropping by 70% (dropping from 1 to 0.3) is taken as the test object. Table 7 shows part of the results of paired T-test, which shows that there are significant differences in the changes of each index value under different failure modes.

Based on the above analysis, we can conclude that when the network is in the growth stage, the random failure mode has the highest robustness, followed by the successive failure of knowledge dissemination behavior mode, successive failure of knowledge contribution behavior mode, collective failure of knowledge dissemination behavior mode, and collective failure of knowledge contribution behavior mode, namely R > BS > WS > BC > WC.

Table 7 shows that the mean values of the differences between indexes in the random failure mode (R) and successive failure of knowledge dissemination behavior mode (BS) are 0.013707 and 0.072419, respectively, which indicates that the range of decline of network performance under these two modes is very close. However, the mean values of the differences between the indexes in the successive failure of knowledge dissemination behavior mode(BS)and the successive failure of knowledge contribution behavior mode(WS)are 0.225036 and 0.214288, respectively, and the mean values of the differences in the collective failure of knowledge dissemination behavior mode (BC) and the collective failure of knowledge contribution behavior mode (WC) are 0.072419 and 0.111128, respectively, which shows that when the network is in the growth stage it is more sensitive to the knowledge contribution behavior failure modes (WS, WC), particularly the collective failure of knowledge contribution behavior mode (WC).Therefore, in this stage, community managers should (a) encourage more collaborative knowledge behavior, (b) give more protection to the main knowledge contributors, and (c) encourage designers who have a high level of professionalism to conduct more knowledge collaboration.

### 5.3. Robustness Analysis during Project Maturation Stage

**Figure 4:**
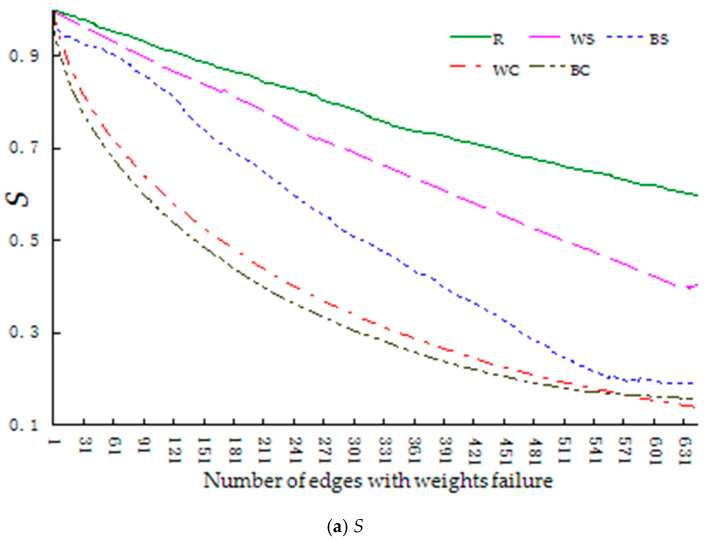

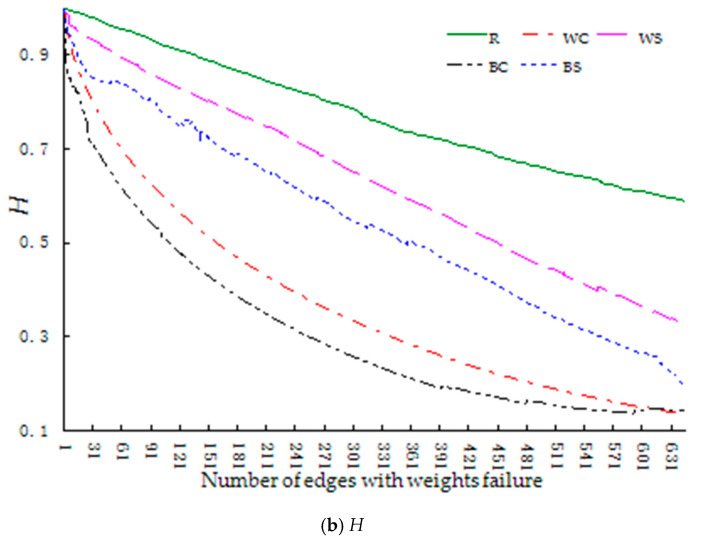
Changes in the robustness index of the network during the maturation stage (Legend number of edges: actual number of edges = 1:5). (**a**) shows the changes in relative size of network connectivity (*S*). (**b**) shows the changes in relative size of collaborative knowledge efficiency (*H*).

The robustness index changes during the maturation stage are shown in Figure 4.

Similar to both the start-up and growth stages, Figure 4 shows that the decline rate of the index value in the maturation stage is higher when the network faces regular failure (BS, BC, WS, WC) rather than random failure (R). Further, the robustness of the network is significantly higher for the successive failure of knowledge dissemination behavior mode (BS) than for the other three regular modes (BC, WS, WC), which differs from the growth stage, and the decline rate of the index value is highest for the collective failure of knowledge dissemination behavior mode (BC). When we performed a paired T-test on this data (with each index value dropping from 1 to 0.3 under different failure modes), the index values of the network under different failure modes were significantly different. See Table 8 for part of these test results.

Based on the above analysis, we can conclude that when the network is in the maturation stage, the random failure mode has the highest robustness, followed by successive failure of knowledge contribution behavior mode, successive failure of knowledge dissemination behavior mode, collective failure of knowledge contribution behavior mode, and collective failure of knowledge dissemination behavior mode, namely R > WS > BS > WC > BC.

Table 8 shows that when the network is in the maturation stage it is more sensitive to the knowledge dissemination behavior failure modes (BS, BC), particularly the collective failure of knowledge dissemination behavior mode (BC).Further, the mean values of the differences between the indexes in the collective failure of knowledge dissemination behavior mode (BC) and the collective failure of knowledge contribution behavior mode (WC) are 0.039459 and 0.051196, respectively, which shows that knowledge dissemination behavior is increasing, that is, many designers are now not only knowledge contributors but also knowledge disseminators. Therefore, in this stage, community managers should try to prevent the collective failure of knowledge dissemination.

Our analysis of the results from the three network stages can be summarized as follows:

1. In the start-up, growth and maturation stages of network evolution, the CKN shows (a) low robustness in the face of regular failure of knowledge contribution behavior and high robustness in the face of irregular failure of knowledge contribution behavior, and (b) low robustness in the face of collective failure of knowledge contribution behavior and high robustness in the face of successive failure of knowledge contribution behavior.
2. During the four regular failure modes (WS, WC, BS, BC), the network’s growth and maturation stages are affected differently. In the growth stage, the robustness of the CKN is lowest during the collective failure of knowledge contribution behavior mode (WC) and highest during the successive failure of knowledge dissemination behavior mode (BS). In the maturation stage, the robustness of the CKN is lowest during the collective failure of knowledge dissemination behavior mode (BC) and highest during the successive failure of knowledge contribution behavior mode (WS).

### 5.4. Robustness Analysis of the Network Facing the Same Behavior Failure Mode in Different Stages

**Figure 5:**
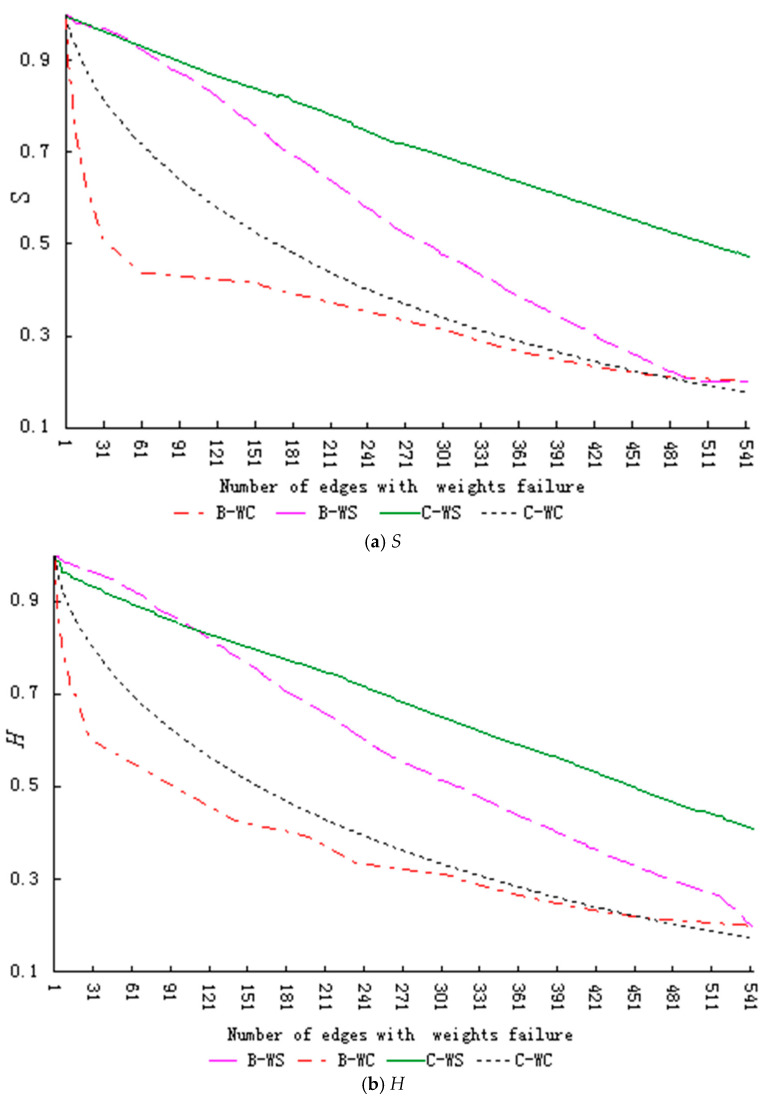
Robustness index values changes of networks in the growth and maturation stages under WC and WS failure modes (Legend number of edges: actual number of edges = 1:5). (**a**) shows the changes in relative size of network connectivity (*S*). (**b**) shows the changes in relative size of collaborative knowledge efficiency (*H*).

**Figure 6:**
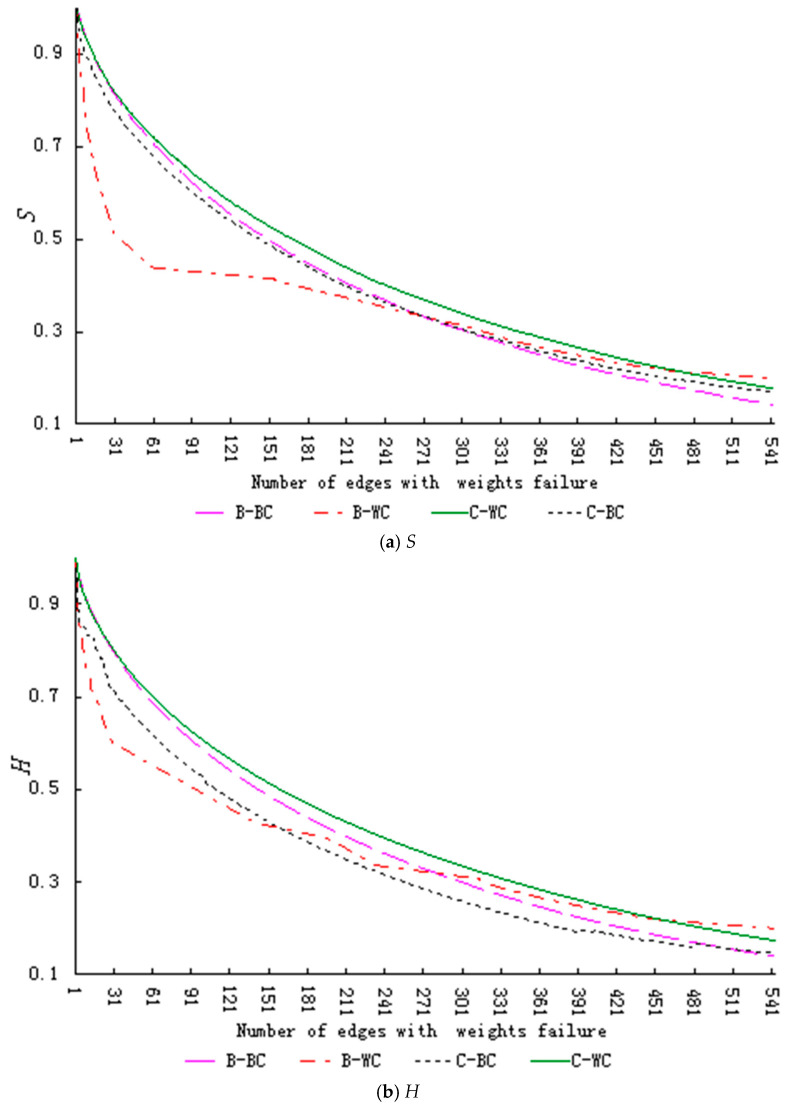
Robustness index values changes of networks in the growth and maturation stages under WC and BC failure modes (Legend number of edges: actual number of edges = 1:5). (**a**) shows the changes in relative size of network connectivity (*S*). (**b**) shows the changes in relative size of collaborative knowledge efficiency (*H*).

To further compare changes in the robustness of CKNs in the evolution process, we analyzed this robustness in different stages of network evolution under the same behavior failure mode. First, we found few cooperative behaviors in the network during the start-up stage, which is very different from the other two stages. The robustness of the network in the start-up stage is obviously lower than that of the other two stages, so we chose only to compare the network in growth stage and maturation stage. Second, as shown in our analysis in Section 5, the robustness of the network is more sensitive when the collaborative behavior collectively fails, and the dynamic evolution of the network under the successive failure mode presents different trends. Third, from a micro perspective, designers reduce the willingness of knowledge collaboration, which is manifests as the collective failure of knowledge collaboration behavior (WC, BC) in the network; however, due to the dynamic evolution of the network, new designers successively enter the community for collaboration, and this manifests in the macro failure of collaborative behavior. Therefore, we compared the robustness of the network in the growth stage and maturation stage under (a) the collective failure of knowledge contribution behavior mode (WC) and successive failure of knowledge contribution behavior mode (WS), and (b) the collective failure of knowledge contribution behavior mode (WC) and the collective failure of knowledge dissemination behavior mode (BC), as shown in Figure 5 and Figure 6, respectively. In these figures, B represents the growth stage network and C represents the maturation stage network.

Figure 5 and Figure 6 show that (a) under the knowledge contribution behavior failure modes (WS, WC), the decline rate of the index value decreases with the evolution of the network, and (b) under the collective failure of knowledge dissemination behavior mode (BC), and the decline rate of the index value increases with the evolution of the network. When we performed a paired T-test on this data (with each index value dropping from 1 to 0.3 under different failure modes), as shown in Table 9, these results show significant differences in the index value of different stages under the same failure mode.

In conclusion, under the collective failure of knowledge contribution behavior mode (WC) and the successive failure of knowledge contribution behavior mode (WS), network robustness is higher in the maturation stage than in the growth stage. However, under the collective failure of knowledge dissemination behavior mode (BC), network robustness is lower in the maturation stage than in the growth stage. This indicates that with the successive evolution of the network and knowledge sharing of designers, the knowledge level of designers in the community is constantly improving. Therefore, more performance can be retained under the knowledge contribution behavior failure modes (WC, WS), which reflects a higher robustness. With the continuous expansion of the community scale, the aggregation coefficient of the network decreases and the weight value of the dissemination behavior increases, which makes the knowledge dissemination behavior have an increasingly important role in the development of the community.

## 6. Conclusions

To explore and improve the robustness of open source projects (OSPs), we constructed a collaborative knowledge network (CKN) based on semantic analysis and edge failure mode. To allow for a more comprehensive study, we used semantic analysis to calculate the edge weight of the network from both the collaborative content intensity and frequency intensity. As this is an edge-weighted network, we carried out systematic robustness analysis experiments from four edge failure modes, to more closely resemble the reality of the project failure process. Our results show that robustness performance varied at different developmental stages of the project: robustness was lowest during the start-up stage and highest during the maturation stage. Further, the robustness of each stage of the project was lower during a collective failure as opposed to a successive failure. Network robustness at the growth stage was most affected by the failure of knowledge contribution behavior, whereas network robustness at the maturation stage was most affected by the failure of knowledge dissemination behavior. These findings prove that it is necessary to analyze dynamic robustness throughout the whole project lifecycle and not just one time period, and community management should focus on different measures to guarantee the robustness of the network. Our research findings led to the following management implications:

1. During the development of an OSP, network robustness trends from weak to strong. Therefore, community managers should pay more attention to network protection during the start-up period of the project. In particular, here it is necessary to introduce enough stable knowledge-contributing designers to avoid the decline of collaborative behavior. For example, in the start-up period of the project, we can invite well-known experts in the industry to participate in the discussion, which can not only improve the cooperation willingness of designers, but also improve the influence of the project, so that more followers can participate in the project cooperation.
2. During the project growth stage, community managers should still focus on the protection of knowledge-contributing designers so as to avoid both the collective decline and successive decline of collaborative behaviors. For example, (1) more resource rights, advanced identity authentication, privilege level and other incentives could be given to designers with major knowledge contribution behavior to encourage them to participate further in knowledge collaboration. (2) Findings Trust, reciprocal benefits and enjoyment are significantly related to positive attitude toward knowledge sharing [52]. Therefore, the community needs to enhance the availability and reliability of the system to improve the collaborative willingness of knowledge contributing designers, For example, setting stars for high-quality knowledge shared by designers, establishing a case database for designers who actively participate in collaboration, and clearly stating the ownership of innovative knowledge in the community and terms of designers.
3. During the maturation stage, more attention should be paid to the protection of knowledge-disseminating designers, including both the collective decline and successive decline of such designers’ cooperative behavior. For example, (1) reward and incentive measures should be given to these designers to increase their sense of belonging and achievement. (2) Enrich the entertainment functions and service functions of the community, such as setting up communication games, answering questions and one-to-one exclusive services, so as to improve the interest and pleasure of knowledge-disseminating.

This paper studies the dynamic robustness of open source design projects by building a semantic based knowledge collaboration network, which is more suitable for large-scale knowledge collaboration as the main way of organization. This kind of community collaboration is more knowledge-based, and the intensity of knowledge collaboration can be more accurately reflected by the content and frequency of collaboration, such as open source product community or open source design community. For other types of virtual communities, such as Wikipedia or social networks, researchers only need to consider the frequency of collaboration to reflect the intensity of collaboration, and then they can build a relatively simple collaboration network and robustness index to study.

Although the semantic-based weighted CKN constructed in this paper incorporates the frequency and content of knowledge collaboration into the calculation of edge weight, which can then describe the CKN more accurately, the directivity of knowledge collaboration has not been considered. Further research could take this into account, and a directed weighted network could be constructed so that more in-depth robustness analysis can be conducted. In addition, the robustness analysis in this paper is based on the single-project network of the OSC. However, in the OSC, the same designer may participate in multiple projects, and any changes in the designer’s collaboration behavior would affect these multiple projects. Therefore, further dynamic robustness research should be conducted on the community’s multi-project CKNs.

## Figures and Tables

**Table 1:** Research on network model and robustness.

Researcher (Time)	Network Model	Network Failure Mode	Robustness Measurement	Dynamic Evolution Stage
Zhou, H.; Zhang, X.; Hu, Y. (2020)	Collaborative knowledge network: user as nodes, frequency of communication as the weight.	Node failure based on: recalculate degree, recalculate betweenness, and random.	The relative size of the largest connected component, et al.	Three different stages of network development (i.e., the start-up, growth and maturation stages)
Martinez-Torres, M.R. (2014)	Weighted network: community members as nodes, number of e-mails as the weight.			One stage
Bellingeri, M.; Cassi, D. (2017)	The co-authorship network of scientists working on network theory and experiment: authors as nodes, the number of common papers and the number of authors of these papers as the weight.	Node failure based on: nearest neighbors (First), next to nearest neighbors (Sec) et al.	The size of the largest connected component (LCC), The weighted efficiency.	
Bai, Y.; Deng, G.S. (2016)	User Interaction weighted network: user as nodes, the number of forwarded interactions between nodes as the weight.			One stage
Duan, D.L.; Lv, C.C.; (2017)	Weighted network	A fraction node failure based on random.	Critical threshold against cascading failures	One stage
He, Z.; Liu, S.; Zhan, M. (2013)	Unweighted heterogeneous networks and weighted heterogeneous networks	Node failure based on: high-degree, low-degree.	The sum of the degrees of inactive nodes	One stage
Frank, S; Pavlin, M. (2020)	Online Social Networks: directed networks of users and their followers (Unweighted)	Node failure based on: the cost-benefit relationship.	Lifetime of the core-periphery structure	One stage
Tanaka, G.; Morino, K.; Aihara, K. (2012)	Coupled oscillator networks: networks consisting of N oscillator nodes coupled by diffusive connections with fixed strength.	Node failure based on: lower degree, random.	Order parameter, Average of the oscillation, Amplitudes over all the oscillators in the phase synchronization state.	One stage
Zhang, X.D; Zhou, H.L. (2017)	Knowledge collaborative network: user as nodes and the collaborative relationship between user as edges	Node failure based on: initial degree, initial betweenness, recalculate degree, recalculate betweenness.	The relative size of the largest connected component, Network efficiency.	Three different stages of network development (i.e., the start-up, growth and maturation stages)
Tang, Y.G; Liao, H.J. (2016)	Regional collaborative innovation network(unweighted network): innovation subject as nodes and the cooperation relationship between innovation subjects as edges	Node failure based on the: comprehensive betweenness.	Average path length, The most connected subgraph.	One stage
Tanizawa, T.; Paul, G.; Havlin, S.; (2006)	Scale-free multimodal network: given values of the number of modes, the total number of nodes, and the average degree	Node failure based on: random, targeted node removal.	Analytical formulas.	One stage
Liu, L.; Meng, K. (2018)	Interdependent networks with correlated structure: two interdependency scenarios: conditional and redundant interaction modes.	Node failure based on: random, low-degree, high-degree.	Giant component size.	One stage
Our work	Semantic-based Collaborative Knowledge Network: designers as nodes, collaborative behavior among designers as edges, and collaborative text content intensity and collaborative frequency intensity as the edge weights.	Edge failure modes based on: (1) collective failure of knowledge contribution behavior, (2) successive failure of knowledge contribution behavior, (3) collective failure of knowledge dissemination behavior, and (4) successive failure of knowledge dissemination behavior.	Relative size of network connectivity, Relative size of collaborative knowledge efficiency.	Three different stages of the project life cycle: the start-up, growth and maturation stages.

**Table 2:** Top ten keywords sorted by weight.

Keyword *t_m_*	Car	Side View	Design	Engine	Track Width	Entry	Package View	Technical	Profile	Rear
weight $\;W_{t_{m}}$	0.998	0.978	0.974	0.969	0.941	0.886	0.883	0.877	0.838	0.801

**Table 3:** Top ten nodes with $g_{i}{}^{\prime }$ value.

**Node** ***i***	328	351	311	325	14	210	317	185	160	356
$g_{i}{}^{\prime }$	1210.57	871.7	811.53	370.21	311.72	267.6	260.6	257.47	218.57	203.53

**Table 4:** Network topology parameters and network characteristics of the semantic-based collaborative knowledge network.

Network	Topological Parameter					Network Characteristic			
	Number of Nodes	Average Out-Degree	Average Path Length	Clustering Coefficient	Network Efficiency	Small World Parameter	Small World Characteristic	Scale Free Property	Assortatvity
Start-up stage	16	1.3529	2.0932	0.1261	0.2151	11.0804	Yes	Yes	No
Growth stage	318	7.9296	2.5445	0.3374	0.2877	19.3845	Yes	Yes	No
Maturation stage	419	7.3430	2.6403	0.3278	0.2630	23.1868	Yes	Yes	No

Note: According to Davis, Yoo and Baker [48], the small-world parameters can be expressed as: SW = [Cactual/Lactual] × [Lrandom/Crandom], where Lrandom = ln(n)/ ln(k),Crandom = k/n, n is the number of nodes, k is the average degree.

**Table 5:** Failure mode design of collaborative knowledge behavior.

	Failure Mode Description	Failure Simulation Calculation Process
Failure Mode of collaborative behavior	Successive failure of knowledge contribution behavior (WS)	Sort the edges generated by the network according to their weights, from large to small, where the weight of the edge with the largest weight is proportionally reduced according to the sorting result. Take the network at this time as the current network, then calculate the weight and sort to reduce the edge with the largest edge weight. Repeat n times to simulate the continuous failure of knowledge contribution behavior.
	Collective failure of knowledge contribution behavior (WC)	Sort the edges generated by the network according to their weights, from large to small. Select the top n weights to connect the edges according to this sorting result, then reduce the weights by a certain percentage to simulate the collective failure of knowledge contribution behavior.
	Successive failure of knowledge dissemination behavior (BS)	Sort the edges generated by the network according to the order of edge betweenness, from large to small, where the edge weight of the edge with the largest edge betweenness is proportionally reduced. Take the network at this time as the current network, then calculate and sort the edge betweenness, where the edge weight with the largest edge betweenness is reduced and repeated N times to simulate the failure of knowledge dissemination behavior.
	Collective failure of knowledge dissemination behavior (BC)	Sort the edges generated by the network according to the order of edge betweenness, from large to small. Select the top n connected edges according to this sorting result, then reduce the weight of edges to simulate the collective failure of knowledge dissemination behavior.
Random failure	Random failure (R)	Randomly select the edge generated by the network according to its weight, where the edge weight is reduced proportionally.

**Table 6:** Paired sample T-test for different failure modes of network in start-up stage (α = 0.05).

	Pairing Failure Mode	M	SD	95% Confidence Interval		t	df	Sig
				Lower Limits	Upper Limits			
	R-BS	0.084231	0.040326	0.067204	0.101262	10.233	23	0.000
*S*	BS-WS	0.153952	0.107466	0.108573	0.199331	7.018	23	0.000
	WS-BC	0.175503	0.058234	0.150913	0.200093	14.764	23	0.000
	BC-WC	0.072582	0.022999	0.062871	0.082294	15.460	23	0.000
	R-BS	0.071423	0.045057	0.052397	0.090449	7.766	23	0.000
*H*	BS-WS	0.161813	0.091040	0.123370	0.200256	8.707	23	0.000
	WS-BC	0.093761	0.038576	0.077471	0.110050	11.907	23	0.000
	BC-WC	0.0804623	0.045413	0.061286	0.099638	8.680	23	0.000

**Table 7:** Paired sample T-test for different failure modes of network in growth stage (*α* = 0.05).

	Pairing Failure Mode	M	SD	95% Confidence Interval		t	df	Sig
				Lower Limits	Upper Limits			
	R-BC	0.013707	0.042816	0.010097	0.01731	7.46	542	0.001
*S*	BC-WS	0.225036	0.135681	0.212829	0.237243	36.224	476	0.000
	WS-BC	0.236496	0.053234	0.230437	0.242554	76.818	298	0.000
	BC-WC	0.072419	0.057574	0.065866	0.078971	21.75	298	0.000
	R-BC	0.006946	0.052469	0.002523	0.011369	3.085	542	0.002
*H*	BC-WS	0.171703	0.125931	0.159653	0.183752	28.009	421	0.000
	WS-BC	0.214288	0.053104	0.208294	0.220281	70.357	303	0.000
	BC-WC	0.111128	0.101701	0.099648	0.122607	19.05	303	0.000

**Table 8:** Paired sample T-test for different failure modes of network in maturation stage (α = 0.05).

	Pairing Failure Mode	M	SD	95% Confidence Interval		t	df	Sig
				Lower Limits	Upper Limits			
	R-WS	0.143179	0.065401	0.138122	0.148236	55.59	644	0.000
*S*	WS-BS	0.086093	0.016223	0.084744	0.087442	125.358	557	0.000
	BS-WC	0.181593	0.061437	0.175039	0.188147	54.501	339	0.000
	WC-BC	0.051196	0.005174	0.080188	0.082204	158.606	254	0.000
	R-WS	0.102239	0.058472	0.097718	0.106760	44.406	644	0.000
*H*	WS-BS	0.133359	0.069257	0.127037	0.139889	41.731	467	0.000
	BS-WC	0.182520	0.048358	0.177392	0.187648	70.003	343	0.000
	WC-BC	0.039459	0.003451	0.039073	0.039848	199.667	304	0.000

**Table 9:** Paired sample T-test of growth and maturation network under the same failure mode (α = 0.05).

	Pairing Failure Mode	M	SD	95% Confidence Interval		t	df	Sig
				Lower Limits	Upper Limits			
	B(WS)-C(WS)	−0.085595	0.073348	−0.092194	−0.07899	−25.487	476	0.000
*S*	B(BC)-C(BC)	0.059745	0.011397	0.058339	0.061151	83.711	254	0.000
	B(WC)-C(WC)	−0.09193	0.049134	−0.09737	−0.086483	−33.207	314	0.000
	B(WS)-C(WS)	−0.134390	0.095798	−0.143556	−0.125223	−28.818	421	0.000
*H*	B(BC)-C(BC)	0.014243	0.012625	0.012818	0.015668	19.670	303	0.000
	B(WC)-C(WC)	−0.13207	0.092625	−0.142322	−0.121818	−25.347	315	0.000

Note: B represents growth stage network, C represents maturation stage network.

## Data Availability

The used and analyzed datasets during the present study are available from the corresponding author on reasonable request.

## Abbreviations

The following abbreviations are used in this manuscript:

CKN	Collaborative knowledge network
OSP	Open source project
OSC	Open source community
WS	Successive failure of knowledge contribution behavior
WC	Collective failure of knowledge contribution behavior
BS	Successive failure of knowledge dissemination behavior
BC	Collective failure of knowledge dissemination behavior
R	Random failure
